# Simulation of COVID-19 Propagation Scenarios in the Madrid Metropolitan Area

**DOI:** 10.3389/fpubh.2021.636023

**Published:** 2021-03-16

**Authors:** David E. Singh, Maria-Cristina Marinescu, Miguel Guzmán-Merino, Christian Durán, Concepción Delgado-Sanz, Diana Gomez-Barroso, Jesus Carretero

**Affiliations:** ^1^Department Computer Science, Universidad Carlos III de Madrid, Leganés, Spain; ^2^Barcelona Supercomputing Center, Barcelona, Spain; ^3^CIBER en Epidemiología y Salud Pública (CIBERESP), Madrid, Spain; ^4^National Centre for Epidemiology, Carlos III Institute of Health, Madrid, Spain

**Keywords:** COVID-19, simulation, social distancing, mitigation policies, face mask

## Abstract

This work presents simulation results for different mitigation and confinement scenarios for the propagation of COVID-19 in the metropolitan area of Madrid. These scenarios were implemented and tested using EpiGraph, an epidemic simulator which has been extended to simulate COVID-19 propagation. EpiGraph implements a social interaction model, which realistically captures a large number of characteristics of individuals and groups, as well as their individual interconnections, which are extracted from connection patterns in social networks. Besides the epidemiological and social interaction components, it also models people's short and long-distance movements as part of a transportation model. These features, together with the capacity to simulate scenarios with millions of individuals and apply different contention and mitigation measures, gives EpiGraph the potential to reproduce the COVID-19 evolution and study medium-term effects of the virus when applying mitigation methods. EpiGraph, obtains closely aligned infected and death curves related to the first wave in the Madrid metropolitan area, achieving similar seroprevalence values. We also show that selective lockdown for people over 60 would reduce the number of deaths. In addition, evaluate the effect of the use of face masks after the first wave, which shows that the percentage of people that comply with mask use is a crucial factor for mitigating the infection's spread.

## 1. Introduction

At the beginning of March, when the number of infections started to escalate sharply in Italy and the first deaths occurred in Europe, the medical community did not yet fully understand the details of how the SARS-CoV-2 virus propagates. A few weeks later, more than 250 million people were in lock-down in Europe; it had started to become clear that this was an exceptional situation. At that point, the need to understand the evolution of the epidemic and the means to contain and mitigate its propagation became a priority for the health authorities. Many researchers started to work on how to better tackle these challenges. EpiGraph (1) is an already existing epidemic simulator that we had developed some years ago and was able to perform large scale, realistic stochastic simulations of the propagation of the influenza virus. During the past months we have adapted our simulator to COVID-19, we added more components, and we increased the detail level and accuracy of the simulations. The current version of EpiGraph has more than 12,000 lines of code written in the C language and parallelized using the MPI library. The simulations we present in this work were executed on the Marenostrum4 supercomputer at the Barcelona Supercomputing Center.

EpiGraph consists of four different modules that work together to capture the transmission between different individuals based on social interconnections, mobility patterns, and climate factors (2). The simulator implements a sophisticated social interaction model, in which the individuals are realistically represented through different characteristics such as age and occupation. Besides the epidemiological and social interaction components, EpiGraph also models people's movements between different urban areas. In this work we have reproduced the mitigation policies taken by the Spanish government and we replicated the behavior of the first infection wave in the Madrid metropolitan area. Starting from this initial scenario, we present an analysis of the effect of potential mitigation policies, such as age-dependent social distancing and mobility restrictions and face mask use.

This work was developed in the context of the project *Medium and Long-term Simulation of Covid-19* funded by the Institute of Health Carlos III, for providing support to the Spanish health authorities, both for the forecast of the current COVID-19 propagation, as well as the evaluation of possible future scenarios. The main contributions of this work are the following:

- We provide a fully detailed description of the EpiGraph simulator and how it is adapted to COVID-19. As part of this, we show how EpiGraph was configured to reproduce the first COVID-19 wave (in Spring of 2020) for the Madrid metropolitan area.
- We evaluate the propagation of the virus under the different mobility restriction policies adopted at different times during the epidemic, including the de-escalation period.
- We analyze the effectiveness of selective social distancing measures and the impact of mask use considering different protection levels.

Section 2 contains a detailed description of the simulator, including its validation comparing both real and simulated values. In section 3, we analyze different mitigation scenarios. Section 4 provides a discussions of the findings as well as the limitations of the work; section 5 describes related work. Finally, section 6 presents the main conclusions of our work.

## 2. Materials and Methods

### 2.1. Background

Algorithm 1 shows an outline of EpiGraph's simulation algorithm. The iterative algorithm discretizes the total simulation time in time steps of 10 min (line 1). In each time step, the algorithm considers each city in the simulated territory (line 2). A city has a given population which is modeled based on the Spanish census data^1^, with the associated social connections between the individuals. Line 5 updates the health status of each infected individual of each city, as indicated by the epidemic model used by the simulator. The next step (line 6) computes how the infectious agent spreads via the social model, starting from every infected individual and evaluating the probability of transmission to each of their contacts. This probability depends on the type of connection, the time of day, and the characteristics of the individual potentially being infected, such as their age or the use of face masks.

**Algorithm 1:** EpiGraph transmission algorithm. Variable *simulation*_*time* represents the simulation duration, *simulated*_*territory* is the simulated area including several cities, each one of them with a social interaction model for the population, and *status* contains characteristics and health status of each individual for each city.


1: **for** *timestep* = 1 → *simulation*_*time* **do**
2: **for** *city* ∈ *simulated*_*territory* **do**
3: **for** *individual* ∈ *city* **do**
4: **if** *status*[*individual*] is *infectious* **then**
5: *UpdateStatus*(*status*[*individual*])
6: *ComputeSpread*(*individual, city*)
7: **end if**
8: **end for**
9: *Individual*_*Interventions*(*status*)
10: *Social*_*Interventions*(*city*)
11: *Transportation*(*city, simulated*_*territory*)
12: **end for**
13: **end for**

We call an individual intervention (line 9) an action taken by the individual to mitigate the propagation of the infectious disease. In Epigraph these actions are activated or deactivated based on defined policies. One example of intervention is that at simulation day 30, a certain individual starts using surgical face masks at work, but not at family time. We call a social intervention (line 10) those interventions—such as school closing or social distancing—that are imposed (or lifted) by the health authorities at a certain time of the simulation. Finally, in the propagation of the infection via the transportation model (line 11), some individuals move between their city and another, depending on the city sizes and the geographical distance between them. This allows us to model the medium and long distance travel of people. The following sections describe each one of these components in greater detail.

### 2.2. Social Model

This section describes how EpiGraph models individuals' characteristics and their social interactions within the region under study. The simulator considers independently every single individual in the population. In this work we simulate the metropolitan area of Madrid with 5,018,241 individuals. In other experiments (not included in this work) we have been able to carry out European-level simulation with up to 198 million inhabitants.

EpiGraph's social model is an agent-based model that captures individual attributes and specifies the way that the individuals interact based on patterns extracted from social networks (Facebook) and from companies (Enron Email Corpus). Attributes include age, gender, and race, which are instantiated based on real census data. We use demographic information to reproduce social habits for four different group types (also called collectives): students, workers, stay-at-home people, and elders. The way the individuals establish social contacts^2^ is time-dependent in order to realistically reflect the temporal nature of the different classes of interactions that each individual has throughout the day. For each one of the group types we consider three different temporal distribution of the individual's activities, those related to weekdays, Saturdays, and holidays (including Sundays).

**Figure 1:**
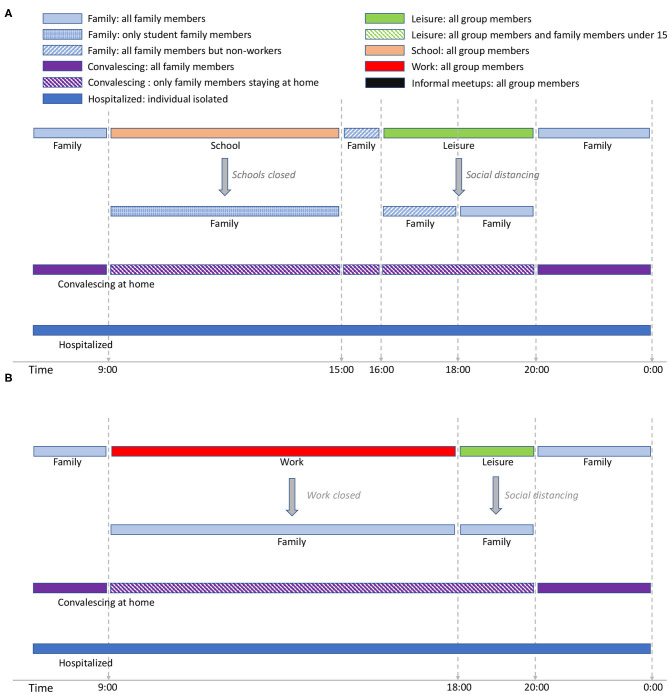
Student activity cycle for weekdays **(A)** and Worker activity cycle for weekdays **(B)**. The interaction patterns are specific for Spain. Night time is considered to extend from midnight to 9:00 and is not included in the figure (but is considered in the simulation). The text in italic shows the effect of social restrictions.

**Figure 2:**
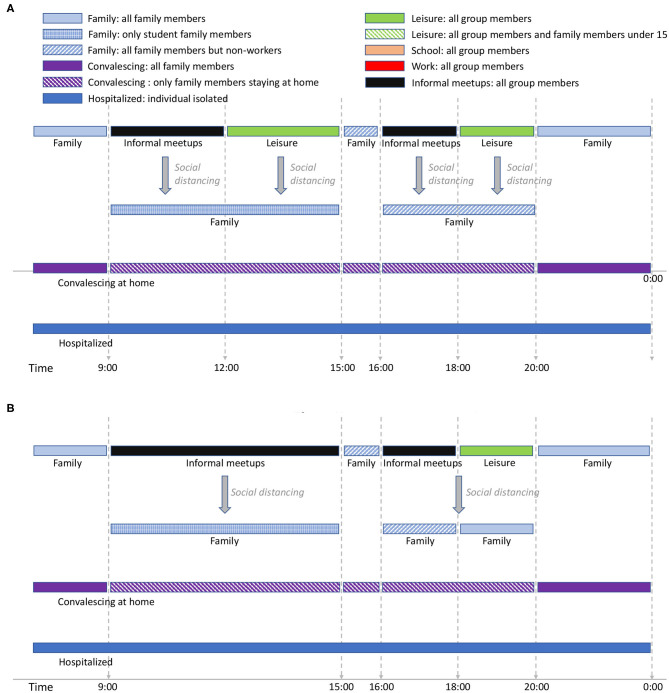
Stay-at-home activity cycle for weekdays **(A)** and Elders' cycle for weekdays **(B)**. The interaction patterns are specific for Spain. Night time is considered to extend from midnight to 9:00 and is not included in the figure (but is considered in the simulation). The text in italic shows the effect of social restrictions.

Figure 1 shows the activity cycles for students and workers during weekdays while Figure 2 shows the activity cycles for stay-at-home and elderly collectives. An activity cycle determines the contacts that are active at a certain time, i.e., the individual interactions with other individuals that may produce a disease transmission. These patterns are specific to the place being modeled; in Spain, for instance, breakfast is around 8:00, lunch time around 14:00, and dinner time starts at 20:00. The period ranging from 0:00 until 9:00 (not shown in the figures) corresponds to family time (i.e., only family connections are active). Note that family time includes all the activities carried out at home (dinner time, family time and night sleep).

For the student group type (Figure 1A), school time is considered to be from 9:00 to 15:00^3^. We assume that school time is followed by a short period of family time, after which there is a leisure period in which the students are in contact with other individuals different from those belonging to the same school group. There are two social distancing policies applied for students: school closure, and social distancing in which school and leisure times are replaced by family time. In EpiGraph, we distinguish between different levels of family-time interactions, based on the family members that are at home at each time of the day, as follows; at night-time each individual is in contact with all family members; when schools are closed, the family-time for this period takes into account only those family members that are at home. For instance, if work places are opened, family time will not include the working members. On the other hand, when social distancing is not imposed, social contacts with stay-at-home and elderly family members are not taken into account during this time period because we assume that these two group types are not at home at this time. For the same reasons, during the family time slot from 15:00 to 16:00, stay-at-home persons and elderly family members are included in the interactions, while working members are not.

EpiGraph can model that a certain percentage of infected individuals stay at home during part of the infectious period. We call this the *convalescing at home* period, in which sick individuals have symptoms that, although not being severe, force them to remain in bed, canceling work, study and social activities. For convalescing individuals, the only contacts are within the family. That is, the infection can only be propagated within the family. In this work we have considered that 20% of the population are convalescing at home after being infected. The simulation also considers individuals with severe symptoms that are hospitalized. In this case, we assume that the patients are isolated in the hospital and do not transmit the disease. The probability of being hospitalized is age-dependent, as described in section 2.4.

The activity cycle for the remaining group types (workers, stay-at-home people, and the elders) are shown in Figures 1B, 2A,B. Each worker has an associated work time slot followed by a short period of leisure time. For stay-at-home individuals and elderly, we define *informal-meetup* as the contacts that a person belonging to these group types creates via typical weekday activities. This includes shopping, retirement home meetings, and social activities related to peer meetings belonging to the same group. In addition, individuals also have leisure periods in which they interact with other groups that may belong to the same or to a different collective.

Supplementary Material includes the activity cycles for Saturdays and Sundays/holidays. For students above 15 years old, we model leisure time on Saturday-night between 20:00 and 0:00. Younger students have assigned family time during this period. In our experiments, 35% of the total of workers work on Saturdays, while the rest don't. For those who do, leisure time ranges from 20:00 to midnight and is shared with the family group. For the stay-at-home and elders' groups there are no contacts within the informal-meetup groups during Saturdays and holidays. We assume that informal-meetup contacts are only related to weekday activities and not performed on weekends.

**Figure 3:**
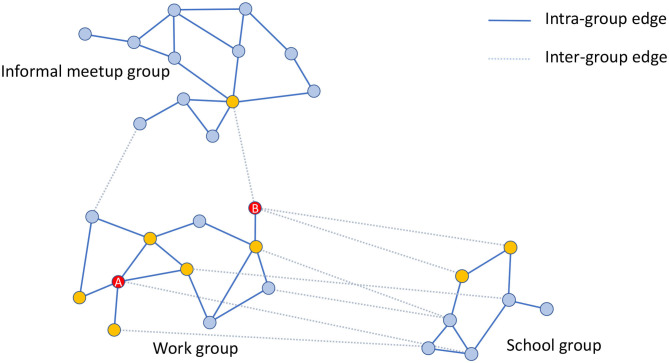
Graphical example of three different groups consisting of 30 individuals in total. The work group consists of 11 individuals that are connected by solid lines that represent the intra-group connections. Yellow nodes are those directly connected with A and B individuals (highlighted in red color) within the same group (work group). The figure also shows inter-group edges represented with dashed lines. These connections are between the work group and the informal meetup and school groups. In this example, work and informal meetup groups are weakly connected whereas work and school groups are strongly connected.

Figure 3 shows an example of a work group consisting of 11 individuals. All the interactions, -denoted as intra-group contacts- and connected by solid lines are between individuals belonging to the same group and occur during the daily activities of this group. The number of interconnections of each individual may be different, e.g., individual A has four connections while B only has one within the same group (work group). This reflects the nature of a real social graph used to generate the groups, where different persons have different connection degrees (3). If individuals A and B are infected, then the people susceptible to being infected within the group will be the nodes displayed in yellow. In this case, individual A has more chances of propagating the disease within the group than individual B. Section 2.4 describes how EpiGraph simulates the propagation of the infectious pathogen throughout the network.

**Figure 4:**
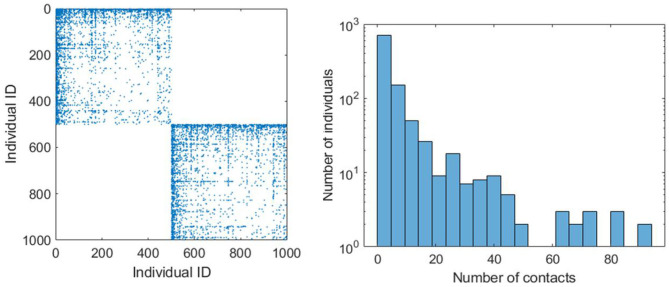
Adjacency matrix (on the left) that provides a graphical example of two connection patterns related to two work groups of 500 individuals. The leisure and family connections are not included. The chart on the right shows the histogram (in logarithmic scale) of the number of individuals that have different number of contacts.

EpiGraph creates different graphs for each work group, school group, stay-at-home (informal meetup) group, and elderly (informal meetups) group. Supplementary Table 1 shows the parameters used for modeling each of these groups. Rather than assuming a distribution or generating synthetic interaction graphs, we use real information from social networks to model the social interaction patterns. Each group has a different size, in between *MinSize* and *MaxSize*. We have used the Enron Email Corpus (70,578 nodes and 312,620 edges) for generating the work and informal-meetup groups while the Facebook (250,000 edges and 3,239,137 edges) network was used to generate the school groups. The adjustment to the desired target size is done using a graph-scaling algorithm based on Random Walk (4). This algorithm selects as many nodes as the group size (i.e., number of individuals in the group) in a random fashion, creating a sampled graph with similar structure to the original one but with a smaller number of nodes. This procedure creates different connection patterns for each group, while maintaining certain graph-related properties such as the distribution of the number of contacts per individual (3). The resulting contact network has an average connectivity of <*k*> = 6.4. This value is obtained according to Equation (1). Where, *N* is the total number of simulated individuals, *K*_*i*,1_, *K*_*i*,2_, and *K*_*i*,3_ represent the number of connections of type 1 (work, school and informal meetups), 2 (leisure) and 3 (family) of each individual *i*, respectively. On the other hand, *P*_*i*,1_, *P*_*i*,2_, and *P*_*i*,3_ represent the duration in hours of each of each connection during a day for individual *i*. Figure 4 shows a graphic example of an adjacency matrix *A* for two groups of 500 individuals each and the histogram (in logarithmic scale) with the distribution of the number of contacts. In this representation, a matrix entry *A*_*i, j*_ ≠ 0 means that individual *i* and *j* have a contact. Note that the family and leisure contacts are not included, thus the figure only shows the contacts within the group. We can observe that the connection pattern is different for each group and that the histogram follows an exponential distribution, which is the usual connection distribution of social networks.


(1) $$\begin{array}{l} <k>=\frac{\sum_{i=1}^{N}K_{i,1}P_{i,1}+K_{i,2}P_{i,2}+K_{i,3}P_{i,3}}{24} \end{array}$$

Existing work such as (5) analyzes the relationship between the structure of the connection network and the propagation of an epidemic, concluding that there exists a direct relationship between the network structure and both the size of the epidemic (as the number of infected individuals) and the timing of the propagation. These findings imply that the use of connection networks based on actual social interactions (3) can contribute to enhancing the simulation accuracy.

The social model includes two more types of social contacts for leisure and family activities. Leisure contacts are modeled by means of inter-group contacts. These contacts are between individuals belonging to different groups (for instance, work and school groups) and occur mostly after the main daily activity and before family time, as well as during the weekends. These contacts represent interactions with friends as well as casual contacts with unknown people. In dashed lines, Figure 3 shows the leisure contacts of the work group with a school and an informal-meetup groups. Note that now, individual B has more inter-group contacts than A. Leisure contacts provide heterogeneity of connections between groups, given that a certain individual can be connected with others belonging to different collectives, for instance, young people with elders, workers with unemployed, etc.

**Figure 5:**
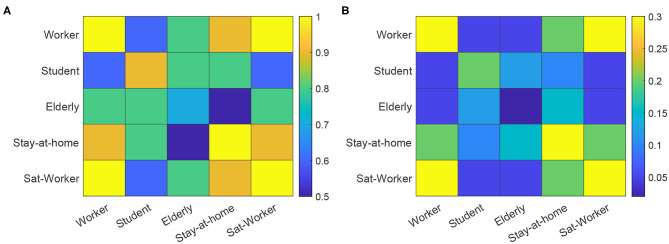
Contact matrices for strong connections **(A)** and weak connections **(B)**. The matrices show the percentage of individuals in the group with smallest number of individuals, that are in contact with the other collective. Sat-worker represents the worker subset that work on Saturdays.

EpiGraph distinguishes two classes of inter-group connections: strong and weak. Groups that are strongly inter-related are tightly coupled, which means that there is a high percentage of individuals that have inter-group connections. This is the case of the work and school groups in the figure. In contrast, weakly inter-related groups, like the work and informal meetup groups in Figure 3, have a small percentage of inter-group connections. This reflects the asymmetry of daily interactions, where some groups (for instance, two different classes sharing the same playground, or two different informal meetup groups sharing the same leisure space) are strongly coupled while in others, that more weakly-related, only few people are involved in the inter-group interactions. The exact percentage of inter-group contacts is given by the contact matrices shown in Figure 5. These matrices show for each pair of collectives, the percentage of individuals that are in contact, either within a strong or a weak inter-relation. For instance, two strongly inter-related work groups will have a large fraction of the individuals with inter-group contacts^4^. Note that, in general, only a small fraction of any group is either strongly or weakly connected. In our experiments, each group is connected strongly with 0.1% and weakly with 2.42% of the total number of existing groups. The inter-related groups are randomly selected, as well as the individuals with inter-group connections.

The third class of contacts are family contacts, interactions with family members who may or may not be part of the same group. The family connections graph is completely connected. However, connections are time-dependent. Supplementary Table 2 shows the distribution of the number of family members, obtained from the INE^1^. Please note that we include single-member family units, where the individual lives alone.

### 2.3. Transportation Model

The transportation model reflects the movement of people between cities for work, study, or vacation, and it is based on the gravity model proposed by Viboud et al. (6). Note that the movement of people within a city is already captured by the social model. The transportation model serves the purpose of moving individuals between different cities, allowing for disease transmission over large areas. The geographical information that EpiGraph takes into account includes latitude, longitude, and distance between urban regions, and was extracted from the Google Maps web service using the Google Distance Matrix API^5^.


(2) $$\begin{array}{l} \left(d_{i,j}<120Km\right)\;\Delta P_{i,j}=\frac{P_{i}^{0.30}Pj^{0.64}}{d_{i,j}^{3.05}} \end{array}$$

(3) $$\begin{array}{l} \left(d_{i,j}\geq 120Km\right)\;\Delta P_{i,j}=\frac{P_{i}^{0.24}Pj^{0.14}}{d_{i,j}^{0.29}} \end{array}$$

This model considers the exchange of individuals between cities, for each pair of cities *i* and *j*. This number (Δ*Pi,j*) depends on the population size in both locations (*P*_*i*_ and *P*_*j*_) as well as the distance between them (*d*_*i,j*_). Equation (2) refers to travel distances of less than 120 Km—which reflects the daily commute of students and workers to neighboring cities. Equation (3) refers to the long-distance commute of workers that need to reside at a different location for several days in a row. Additionally, we consider people from any group type that move at any distance for several days for vacation purposes. Once the volume of inter-city commuters is calculated, we randomly select individuals from specific group types within the populations and move them for a specific period of time to other locations. In our experiments, for the short distance commuters, 85% are workers and 15% are students; for the long-distance commuters the percentages are 50% workers, 30% students, 15% retired individuals, and 5% unemployed people.

### 2.4. COVID-19 Model

**Figure 6:**
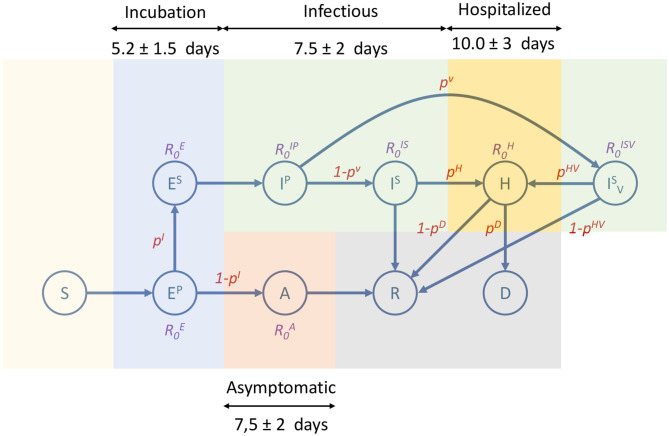
Compartmental model used by EpiGraph. It consists of the following states: susceptible (*S*), primary exposed (*E*^*P*^), secondary exposed (*E*^*S*^), asymptomatic (*A*), primary infectee (*I*^*P*^), secondary infectee with antiviral treatment ($I_{V}^{S}$), hospitalized (*H*), recovered (*R*) and dead (*D*). Each state shows the basic reproduction number of the state (non-existing R0s means that are not applicable). The edges show the transition probabilities (which are normalized) between the compartments. Duration of the main infection stages consists of an incubation that includes *E*^*P*^ and *E*^*S*^; infectious includes *I*^*P*^, *I*^*S*^, and $I_{V}^{S}$ (which is not considered in our experiments); hospitalized is represented as *H*; and asymptomatic is *A*. Note that the asymptomatic stage starts after the primary exposed stage (*E*^*P*^), which in this approximation lasts only 1 day.

The epidemic model implemented in EpiGraph is a compartmental stochastic SEIR model extended to include compartments for incubation, asymptomatic, and dead, as well as an additional hospitalized state. However, instead of being an analytic model based on differential equations, Epigraph follows an approach based on probabilities using randomness to determine the duration and transitions between the compartments. In addition, the basic reproduction numbers *R*_0_*s* are different for each compartment. Figure 6 shows the infection phases, which are described below; Table 1 shows the *R*_0_ values for each compartment, as well as the transition probabilities for the compartments that can transit to different states (like *E*^*P*^). The different infection stages are:

- **Incubation stage**. At the beginning of this stage individuals are infected but symptoms are not present and they are not yet able to transmit the virus. This stage is represented as primary exposed *E*^*P*^. From this stage the infection can enter one of two phases, based on a probability *P*^*EI*^: a secondary exposed stage *E*^*S*^ where slight symptoms appear and the individual becomes infectious with a certain $R_{0}^{ES}$, or an asymptomatic stage (described below). We assume that $R_{0}^{ES}$ is the same as the asymptomatic $R_{0}^{A}$.
- In the **asymptomatic stage** (compartment *A*), infected individuals do not notice symptoms but are able to transmit the disease with a certain $R_{0}^{A}$ reproduction number. After a certain time, they pass to the recovered compartment in which the subject acquires viral immunity.
- In the first **symptomatic stage**—called primary infection state *I*^*P*^—symptoms appear and a certain fraction of the individuals (given by a probability *P*^*V*^) seek medical attention. This may imply initiating antiviral therapy ($I_{V}^{S}$ state) —which is not considered in this work. In our experiments, all infected individuals will transition from *I*^*P*^ to *I*^*S*^. In addition, *I*^*P*^, *I*^*S*^, and *I*^*V*^ have associated basic reproduction numbers of $R_{0}^{IP}$, $R_{0}^{IS}$, and $R_{0}^{IV}$.
- A certain fraction of the individuals are hospitalized (**hospitalized stage**). The probability of entering this stage is given by the parameter *P*^*H*^(*age*), which is age-dependent. Table 2 shows the values for *P*^*H*^(*age*), obtained from (11). Note that this probability increases with age. From this state, an individual may transition to either the recovered or the dead stage. During hospitalization, we use $R_{0}^{H}$ for modeling the transmission in hospitals. In this work we assume that due to the controlled conditions of hospitalized individuals, the transmission risk is reduced to 10% compared to a non-hospitalized person, and we use $R_{0}^{H}=0.34$ as a result. For the purpose of this work, we assume that a recovered individual acquires indefinite immunity to the virus.
- The individuals that reach the **dead stage** are removed from the simulation. The transition probability, denoted as *P*^*D*^(*age*), is also age-dependent. Table 2 shows these probabilities, which have been obtained from (11). Note that this probability is applied over the portion of hospitalized individuals.

**Table 1:** *R*_0_ Values and transition probabilities for each compartment state.

**Compartment state**	**R**_**0**_ **values**		**References**	**Probability**	
*E* ^*P*^	$R_{0}^{EP}$	0		*P* ^*A*^	25%
*E* ^*S*^	$R_{O}^{ES}$	1.42		N/A	
*A*	$R_{O}^{A}$	1.42	(7)	N/A	
*I* ^*P*^	$R_{O}^{IP}$	4.5	(8)	*P* ^*IS*^	100%
*I* ^*S*^	$R_{O}^{IS}$	3.38	(9, 10)	*P* ^*H*^	Table 2
$I_{V}^{S}$	$R_{0}^{ISV}$	N/A		N/A	
*H*	$R_{0}^{H}$	0.34		*P* ^*D*^	Table 2

*In this work we have not considered the use of antivirals, thus $I_{V}^{S}$ state is not reached and the associated $R_{0}^{ISV}$ value is not applicable. E^S^ and A states do not have a related transition probability because there is only a destination state*.

**Table 2:** Values of *P*^*H*^ and *P*^*D*^ based on age.

	**Age interval**								
	**<10**	**10–19**	**20–29**	**30–39**	**40–49**	**50–59**	**60–69**	**70-79**	**≥80**
*P*^*H*^ (%)	0.4	0.4	3.4	9.0	19.6	31.4	40.8	49.8	45.2
*P*^*D*^ (%)	0.0	0.4	0.8	0.8	1.2	2.0	4.7	12.2	30.0

*P^H^ is the probability an infected person has of becoming hospitalized and P^D^ is the probability a hospitalized person (a fraction of the total infected) of dying*.

The time spent in a given state is generated following a normal distribution to simulate the time ranges specific to each stage of the infection and the fact that each individual may go through phases of different lengths. Figure 6 shows an overview of the different infection stages. We also consider that a percentage of the sick individuals stay in bed, thus reducing the number of people that they interact with.

While the EpiGraph model implements all the necessary phases and variables, it needs to be fine-tuned for COVID-19. We adopt most of the concrete values for the model parameters from the existing literature. More specifically, (12) reports on an incubation period of 5 in mean. We considered that of these, 3 days correspond to the primary and 2 days to the secondary incubation phases (13). The difference between these phases is that in the primary phase there is no risk of transmitting the disease. We used a normal distribution to associate a different stage duration to each infected individual. We took the standard deviation for the incubation period to be 1.5 days, and we distribute it proportionally between stages. We assume that the related $R_{O}^{ES}$ is the same as the asymptomatic $R_{O}^{A}$ (described below).

We adopt an average infectious period of 5.90 days; a similar value to that reported in (14). This period is divided into two phases: the primary infection period that we assume to last for only 1 day, while the secondary infection lasts the remaining 4.9 days (14). We adopt a standard deviation for the infectious period of 2 days, which was proportionally distributed between the first and second periods. We found studies that pointed to higher virulence at infection onset, which we understood as the primary infectious stage. We therefore take the higher number in the literature (4.5) as the $R_{O}^{IP}$ at the onset of the infection (8), i.e., for the primary infectious stage. For the rest of the period, i.e., the secondary infection period, R0 is taken to be 3.38 (9, 10).

Based on a study of the Diamond Princess cruise ship (15), 322 of 621 people on board tested positive but showed no symptoms. Given the controlled environment and fact that it was easy to test the passengers in their entirety, we believe that the percentage of people that go from incubation to asymptomatic is actually quite large. For lack of other data, we set it to roughly half the percentage on this cruise ship. This is also coherent with the ECDC reported values (16). Consequently, we assume that 25% of the infected individuals become asymptomatic. We used an asymptomatic phase duration of 5 days (13). According to (7), asymptomatic individuals were 42% less likely to transmit the virus than symptomatic people. Consequently, we take this basic reproductive number $R_{O}^{A}$ to be 42% of $R_{O}^{IS}$.

In (17, 18), it is mentioned that the median number of days from first symptoms to death was 13. Given that the average duration of the *E*^*S*^, *I*^*P*^, and *I*^*S*^ stages is 8 days^6^ and an individual only dies after a hospitalization period, we adopt the mean time of being in hospital of 5 days. The simulator evaluates the risk of being hospitalized and dying based on the age of each individual. The age distribution among the existing group types was extracted from the INE (the Spanish National Statistical Institute).

### 2.5. COVID-19 Mitigation Strategies

Given that the simulator considers every single individual and their connections, it is possible to model in detail the different social distancing and mitigation policies imposed by the authorities. We developed a new component called the *mitigation model* that links these policies with the social and the mobility models. The policies that we are considering are:

- Social distancing. EpiGraph distinguishes four classes of contacts between individuals: at school, at work, with family, and during leisure time. We leverage this distinction to evaluate social distancing policies that can apply differently to the contact types, for instance the closure of the schools and work places. We have considered both essential and non-essential workers—which represent 35 and 65% of company employees—as well as the interruption of leisure activities.
- Mobility restrictions. The transportation model of EpiGraph includes long and short-distance movements of individuals between cities. In this work, we have introduced policies that restrict each one of them independently.
- Face-masks. We have introduced the use of surgical and ffp2-grade face-masks, which we evaluate when used by the general population or by targeted groups, such as the elderly.

### 2.6. Setting Up the Simulator Configuration

For the experiments presented in this work, we focus on results for the metropolitan area of Madrid, which includes the city of Madrid and the following surrounding satellite cities: Alcalá de Henares, Alcobendas, Alcorcón, Fuenlabrada, Getafe, Leganés, Móstoles and Parla—for a total of 5,018,241 inhabitants according to the census data^1^. The baseline scenario we modeled reproduces the social distancing measures that were applied in the Madrid metropolitan area in Spring of 2020. The simulation starts on March 3rd with a certain percentage of infected individuals, rather than with a patient zero. In our experiments, 0.6% of the population of each city was initially infected at this time. The lockdown occurred on simulation day 13 (equivalent to March 16th), when partial enforcement policies were applied, which include school closure, working from home for 65% percent of the businesses, social distancing (where all leisure connections are disabled), and travel restrictions—all reflecting the real-life policies that were enforced in Spain on that very date. On week before lockdown (March 9th) we introduced partial social distancing with a reduction in the number and duration of leisure contacts. This reflects the existing change in the behavior of the population just before lockdown. On simulation day 27 (which corresponds to the 30th of March), 100% of the companies closed or instated 100% work-from-home policies. At this point only the family connections are active. Finally, on day 41 (13rd of April), 35% of the companies reopen, activating the corresponding work connections. In this baseline scenario the rest of the population remains confined indefinitely in order to avoid creating a second wave of infection.

EpiGraph has to be initially calibrated to reproduce precisely the COVID19 spread. For each contact between a pair of infected and susceptible individuals, the transmission probability depends on the infected individual's R0 value in the current infection phase and the duration of the contact. We have used a scale factor that increases or decreases this probability in order to produce realistic infection spreads. In total, three configuration parameters need to be specified: the scale factor, the initial number of infected individuals and the simulation starting time. In this work, the calibration was performed using the first wave in Spain. The goal was to replicate the shapes of the curves using the distributions of the number of daily infections and deaths (see details below), as well as to achieve the same prevalence value as the one in Spain after this first wave. After the calibration process the best fitting values were 7.14 for the scale factor, 0.6% for the initial percentage of infected population and March 3rd for the simulation start time. Note that these parameters are constant and will be used in all the experiments shown in this paper.

In the simulation outcome, and average percentage of 12.2% of the population becomes infected in the period until the simulation ends (on June 26th), a percentage similar to the one obtained from the prevalence study carried out in Spain (19), which predicts a prevalence of 11.7% for the metropolitan area of Madrid at this time, and a 5% as the average value for the country.

**Figure 7:**
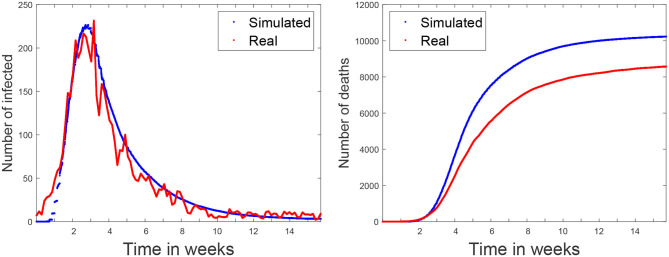
Baseline scenario. simulated and real data of the COVID-19 spread in Madrid metropolitan area for Spring 2020. Simulation starts on March 3rd. The figure on the left shows the number of infected per time step (10 min). The figure on the right shows the accumulated number of deaths.

Figure 7 (left) shows both the real (in red) and simulated (in blue) distributions of infected cases. The simulated values are the average of five independent simulation and have been scaled to the current population of the community (6.6 million, which includes the inhabitants of other smaller urban and rural areas). The daily reported cases of infections were obtained from^7^. Our aim is to compare the temporal distribution of infections for the reported and simulated cases. Note that the reported cases do not precisely reflect the actual number of infected individuals at a certain time, given that an important fraction of the cases are unaccounted for, including most of the asymptomatic cases and the unreported infections. In order to compare the two curves, in Figure 7 (left) we scaled each one according to their prevalence percentages (11.7 and 12.2% for the real and simulated distributions, respectively) on June 26th (which corresponds to the end of the simulated scenario). The area under the red curve corresponds to the 11.7% of Madrid's population, while the area under the blue curve represents 12.2% of the population^8^. We can observe that the curves have a similar shape, with a rapid initial increase in the number of cases and a long decreasing infection tail.

The last validation was carried out using the number of officially reported deaths. Figure 7 (right) shows with a red line the accumulated official number of deaths^7^. Note that, due to the uncertainly of the data, it is impossible to know if all the COVID19 deaths have been reported. For this reason, this curve represents a lower bound of the deaths. The blue line represents the simulated values that are the average of five independent simulations. According to (17, 18), the average time between the first symptoms and death is 13 days, which is the same that the simulations takes to go through the incubation, infectious, and hospitalized stages (see Figure 6). Empirically, the time difference between the simulated and the real deaths is 7 days (in the figure the simulated values have been already shifted by this value). This discrepancy is likely related to delays in the reporting of the deaths.

**Figure 8:**
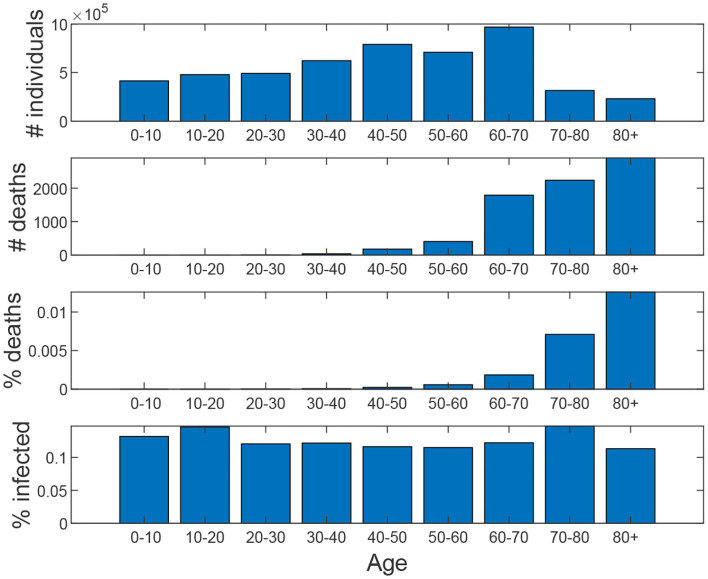
Baseline scenario. From top to down: age distribution of the population, age distribution of the number of deaths, percentage of deaths for each age interval, percentage of infected for each age interval.

Figure 8 shows the age distribution of the simulated population as well as the distribution of infection and death cases. We can observe that the elderly suffer a higher death toll, as expected.

## 3. Results

In this section, we present results for different scenarios, including different lockdown alternatives, the impact of the use of face masks, social distancing measures, and the use of testing for the quarantine of early-detected infected individuals. All experiments start on the 7th of March with 0.01% of the population being infected; we apply the calibration described in section 2.6. The simulation captures as infected individuals not only the reported cases, but also those individuals that are not reported, as well as the asymptomatic cases.

On 18th of May, Madrid entered Phase 0.5 and most of the lockdown restriction were lifted. The first alternative scenario represents the worst-case scenario, in which at this time (simulation week 11) all the restrictions are lifted, including social distancing measures and travel restrictions, and schools open. No masks were used at this time.

The idea of this scenario is to evaluate the maximum virus spread. **Figure 11A** shows that under these conditions, 95.7% of the population becomes eventually infected, and the death toll rises to 70,000. In this scenario, the effect of school closure on the disease propagation is minimal; we have simulated the same scenario keeping schools closed, while the rest of the restrictions are lifted, and 93.5% of the population becomes infected.

### 3.1. Evaluating Face Mask Effectiveness

In this section, we evaluate the mitigation capabilities of different types of face masks, while considering different percentages of the population that use each type. The effectiveness of masks and face covers remains uncertain and depends on diverse and complex aspects including, among others, the ability to reduce the outward particle emission rates for the different transmission modes (droplet spray or aerosol) (20), the way this effectiveness is degraded after using or washing the mask, and the lack of experience in using them correctly, all of them resulting in increasing the risk of infection. EpiGraph models face mask effectiveness as a scale factor over the probability of transmitting the infection. For example, an effectiveness of 90% means that the probability of contagion using masks is reduced to 10% with respect to the case of not using them. Note that the way we define mask effectiveness is different from that of existing studies: instead of representing the capability of the mask to prevent the infection, we consider its effectiveness as a whole, which includes not only the protection provided by the mask by itself but also the change that taking this measure triggers in the individual habits; for instance, keeping a safe security distance with other people or the use of hygienization measures, e.g., hand washing or using hydro-alcoholic gels after contact with others. With this definition, we believe that the actual effectiveness provided by a mask may be larger than the actual reported one.

**Figure 9:**
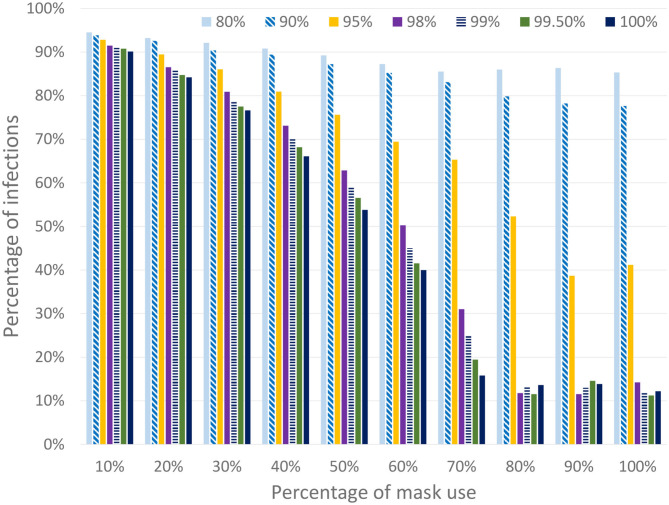
Percentage of infected individuals for different percentages of people using masks and different mask effectiveness. The simulated scenario considers that all work and leisure restrictions are lifted on the 18th of May (while schools remain closed).

For the sake of simplicity, and in order to focus on the effect of face masks, all the scenarios in this section use the worst-case baseline shown in **Figure 11**, in which all the work and leisure restrictions are lifted on the 18th of May. Note that this scenario is similar to the existing condition in Spain at this time. At this time we assume that a certain percentage of the population starts using masks during outdoor activities but not during family time (which is to be expected), thus the transmission risk between cohabiting individuals (i.e., family contacts) is not avoided. EpiGraph considers distinct effectiveness with regard to preventing an infected individual from transmitting the disease or a susceptible individual from becoming infected. In this first part of the experiments we assume that both effectiveness are the same, thus the mask protects both infected and susceptible individuals. Figure 9 shows the final number of infected individuals for different usage percentages (x-axis) and mask effectiveness. We can observe that both parameters are strongly related with the infection spread. EpiGraph performs the simulations using a stochastic approach in which the results of different executions may differ. This may create small fluctuations in the results. We think that these fluctuations are the reason of having non-monotonic decreasing values when the percentage of mask use is larger than 70% and the mask effectiveness are larger than 95%.

**Figure 10:**
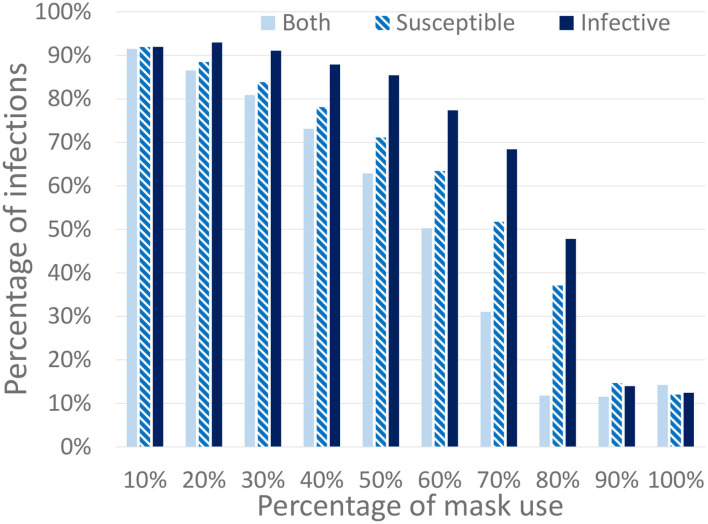
Percentage of infected individuals for different percentages of the population using masks. We consider three different mask protection levels: protecting the infected, the susceptible, or both of them. All work and leisure restrictions are lifted on the 18th of May (schools remain closed).

The second study that we have performed, shown in Figure 10, consists of three scenarios that distinguish the mask protection between infected and susceptible individuals and considers different mask effectiveness. In the first one, denoted that the mask protects both of them, is similar to the previous ones where the masks have the same effectiveness for both infected and susceptible individuals. In the second one (where the mask protects the susceptible individuals) we consider that the masks are only effective for susceptible individuals. In this case, a mask does not reduce the transmission risk from the infected individual but protects a susceptible from being infected. In the third scenario (where the mask protects the infected individuals) the opposite is true: the mask prevents the transmission from infected individuals but it does not protect the susceptible. Note that these hypotheses, although unrealistic, are included as limits to represent the most extreme scenarios where the protection is completely biased to only infected or only susceptible individuals. A real mask would produce an intermediate protection between these limits. In the figure, we can observe that these scenarios produce a significant decrease in the final number of infected individuals.

### 3.2. Policies Targeting the Elderly

The mobility strategies we consider relate to different work and free movement restrictions for people above 65 years of age, school closing, and travel restrictions. We refer to people above 65 as the elderly. Given that this group has the highest mortality levels, in this section we evaluate the effectiveness of specific targeted lockdown policies. In order to simplify the analysis, the use of face masks is not initially considered in this section.

**Figure 11:**
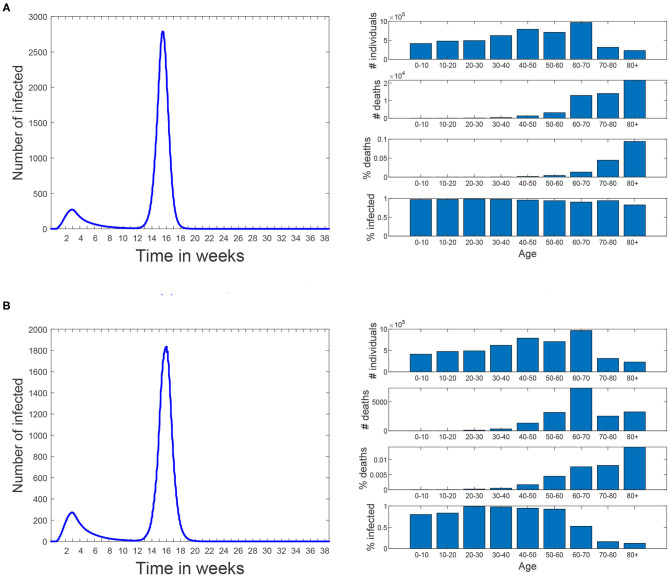
Scenarios 1 and 2. In both of them simulation starts on March 3rd and the same restrictions than the baseline scenario are applied until 18th of May. Since then all restrictions are lifted until the end of the simulation. No masks are used. **(A)** Scenario 1: all the restrictions are lifted for all the population. **(B)** Scenario 2: people over 60 are kept isolated since March 16th until the end of the simulation.

Scenario 2 is an ideal (and rather unrealistic) case in which the elderly keep social distancing indefinitely since March 16th. This is defined as disabling contacts of all types, including with their families. Starting from the baseline scenario, work and free movement restrictions are lifted for people under 60 on 18th of May. Schools remain closed and travel restrictions are maintained. Figure 11B shows the simulation results. Note that the number of infected individuals is slightly reduced when compared to the first scenario (now is 76%) but the total number of deaths is reduced to nearly one third (23,800 deaths). The reason is that the most vulnerable population group is prevented from getting infected, thus reducing the number of hospitalized and death cases.

Scenario 3 reflects a less restricted case, in which the elderly keep social distancing until simulation week 20 (17th of July). At this time they are allowed to return to normal life using mask with a protection of 99.8%. The aim of this scenario is to isolate the effect of the confinement of the elderly, while the rest of the population does not take any special measures to avoid transmission. Results show the same percentage of infections and 23,900 that the number of infections does not increase significantly due to herd immunity being already reached at the time the confinement of the elderly ends. Note that in these experiments we assume that a recovered individual acquires indefinite immunity to the virus and that the virus does not mutate during the simulation time.

Scenario 4 is a variation of Scenario 3 in which the elderly keep social distancing until the 18th of May (instead of the 17th of July). From this date on, they use masks with a protection of 99.8% at all times except during family connections. In this scenario, the number of infections increases to 88.7% and the deaths to 57,000. Note that, despite of mask use by the elderly, now they are not isolated during the second infection peak an thus suffer a significant number of deaths.

**Figure 12:**
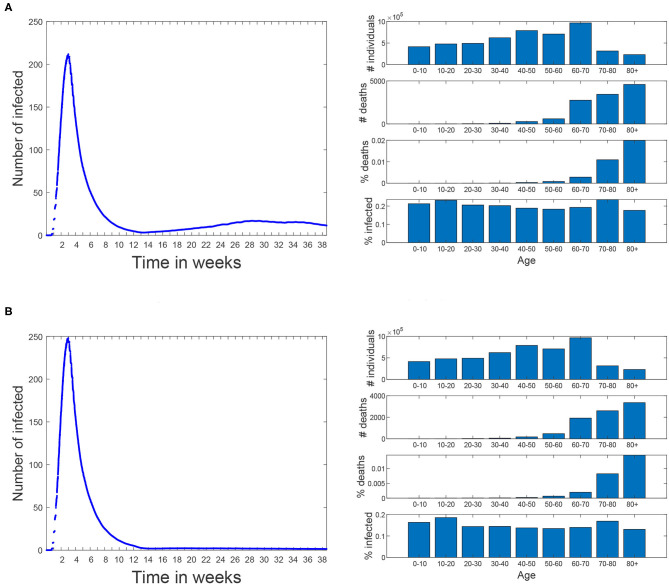
Scenarios 5 and 6. In both of them simulation starts on March 3rd and the same restrictions than the baseline scenario are applied until 18th of May. Since then, all restrictions are lifted until the end of the simulation. Since 18th of May 70% of the population (piked at random) start using masks with an effectiveness of 99.5%. **(A)** Scenario 5: all the restrictions are lifted for all the population. **(B)** Scenario 6: people over 60 avoid any social contacts but keep the familiar ones.

In order to represent a more realistic scenario we have modeled Scenario 5 (shown in Figure 12A) in which 70% of the population (picked at random) uses masks with an effectiveness of 99.5%. In this scenario a second infection wave occurs and, at the end of the simulation (week 38 corresponding to November 24th), 19.0% of the population is infected and results in 15,000 deaths. Note that the infection percentage is similar to the prevalence study for Madrid (21), which rounds 18.6% of Madrid population and that was carried out between the 16th and the 29th of November. The aim of this scenario is not to precisely reproduce the second infection wave in Madrid, but rather to evaluate different policies under a similar percentage of infections as the real one.

Scenario 6 (shown in Figure 12B) corresponds to a variation of Scenario 5 where people over 60 restrict themselves from all contacts but the familiar ones (where no masks are used). In this case the percentage of infections is reduced to 16.0% and the number of deaths is reduced in a greater proportion, all the way down to 11,600.

The last scenario (Scenario 7) has the same social distancing restrictions for the elderly as Scenario 6. In addition, workers over a certain age threshold are isolated from all contacts but the familiar ones. When this threshold was set to 60 years, the percentage of infections and number of deaths is similar to Scenario 6 (15.7% and 11,400), but when this threshold is reduced to 50 years, these values are reduced to 12.9% and 10,900.

## 4. Discussion

This work presents the first results of the simulations using EpiGraph, for the Madrid metropolitan area and the first COVID-19 wave of Spring 2020. After creating an accurate social model of Madrid and reproducing the same mitigation measures as those taken by the authorities we first validated the simulator by comparing the predictions with the real values. Here we faced the problem related to the uncertainly of the real data, which is based on reported cases. Given that the reported cases are only a fraction of the real ones and that it is impossible to determine precisely how big this fraction is, we first compared the final number of infected individuals with the seroprevalence study provided in (22). These values were similar enough, at 15 vs. 12%. The second validation was comparing the number of reported and simulated deaths (shown in Figure 8). Again, here there is also an uncertainly because the reported death cases related to COVID-19 that are provided by the health authorities smaller that the total number of deaths reported by the civil registry. Here, the simulated values are higher than the reported cases.

Once the validation was completed, we used the simulator to evaluate different scenarios after the real lockdown situation of the region and related to social distancing of the elderly and the impact of masks use. The information provided by these simulations represent a two-fold contribution. On the one hand, they were used to assess different social distancing policies; on the other hand, they evaluate different protection levels of mask use among different percentages of the population. In total, both of them provide examples of the simulator features.

EpiGraph is a thoroughly validated simulator that models in detail most of the important factors in the propagation of an epidemic. It does nevertheless have certain limitations, some that have to do with the modeling of a new virus which is not 100% understood. The first limitation is that in the current version of EpiGraph, a recovered individual acquires indefinite immunity to the virus, which makes it impossible to be re-infected for the duration of the simulation. If the simulated time is long, this assumption may no longer hold. We are currently working on implementing this feature in the simulator.

Another limitation is that the current transportation model is a gravity model based only on the distance between the cities and the population size. Having real knowledge about mobility patterns, for instance about those individuals using public transportation means, would provide a much more realistic approximation than the gravity model. It is also worth to mention that in the context of this work (with a prolonged lockdown) the influence of the transportation is reduced.

We currently assume 25% percent of the infected people to be asymptomatic. Research such as (23) assumes that the transmission rate of the virus is constant during the exponentially growing phase, and they use a time-dependent exponentially decreasing transmission rate to model the change in R0 after the early exponentially increasing phase. Theirs is an attempt to examine the number of asymptomatic infectious cases and unreported infectious cases; this type of approximation may be useful to perform a more precise calibration of the simulator. A last limitation is that EpiGraph uses contact matrices based on group types. We are currently working on a more detailed model based on contact matrices for age ranges, which will increase the accuracy of the social model.

Finally, it is necessary to refine the social model in order to include different collectives, among others, health and social-health workers and elderly people at nursing homes. This would produce more realistic forecasts. In this work, health care facilities are modeled as informal meetup groups for the elderly, which allow social gatherings. Besides that, leisure contacts also provide heterogeneity by allowing contacts with different collectives in which individuals may belong to different age groups.

## 5. Related work

There are a vast number of publications on Covid since the beginning of the pandemic, a lot of them treating the same problem as we do—simulating the propagation of the virus under different scenarios of intervention on part of the governments. Friedman et al. (24) identifies 383 published or publicly released COVID-19 forecasting models.

DELPHI (Differential Equations Leads to Predictions of Hospitalizations and Infections) (25) is a compartmental model that is based on the widely successful SEIR model, with additional features such as modeling under-detected cases and governmental response measures. To model the response, the authors multiply an initial infection rate with an arc tan curve, along with an exponential jump correction to model the resurgence in cases in many places. They model the potential impact of various policies on future infections by estimating the average effect of each measure as implemented across states, via training.

Youyang Gu‘s COVID-19 model (26) applies machine learning to derive the basic reproduction number (R0) from data published by Johns Hopkins University’s Center for Systems Science and Engineering (CSSE), and hooks this to a compartmental model. Their infection estimates include all infected individuals of the SARS-CoV-2 virus, not just those that took a COVID-19 test and tested positive.

The COFFEE model from Los Alamos National Laboratory (27) produces “forecasts, not projections; meaning it does not explicitly model the effects of interventions or other ‘what-if’ scenarios. We distinguish forecasts as attempts to predict what will happen vs. projections as attempts to describe what would happen given certain hypotheses.” COFFEE is probabilistic and it is fit to geographic regions independently, facilitating parallelization for fast computations. The method fits weighted regressions to the training data and compute Joint Probability Distributions over tuning parameters.

Imperial College London has several planning tools in place. Of these, Flaxman et al. (28) describes an extension of a semi-mechanistic Bayesian hierarchical model that infers the impact of interventions and estimates the number of infections over time. This approach works under the assumption that changes in the reproductive number are an immediate response to interventions rather than broader gradual changes in behavior, and are calculated backward from temporal data. The authors use the discrete renewal equation as a incubation process for the modeling of infections and propose a generative mechanism to connect infections to death data. They use this joint Bayesian hierarchical model to produce short-term predictions, and they apply their model to 11 different countries.

In (29), the authors use a deterministic SEIR framework to model the propagation of the virus and the effect of non-pharmaceutical interventions (social distancing mandates and mask use) until the Spring of 2021. The model also uses projections of pneumonia seasonality, mobility, testing rates, and mask use per capita to predict infections, deaths, and hospital demand. In terms of social distancing, they include the following measures: (1) severe travel restrictions, (2) closing of public educational facilities, (3) closure of non-essential businesses, (4) stay-at-home orders, and (5) restrictions on gathering size. Based on data from Facebook, Google, SafeGraph, and Descartes Labs, the authors use a Bayesian, hierarchical meta-regression model with random effects by location to approximate the expected change in mobility. Based on data from February to September, they fit relationships between changes in the rates at which infectious individuals may come into contact and infect susceptible individuals and mobility, testing, masks, pneumonia seasonality and others. Some of the limitations of this approach are the exclusion of movement between locations, the absence of age structure and mixing within location (assumption of a well-mixed population), and the inability to model super-spreader-like events.

In (30), the authors focus on a better description of sojourn time, the duration before clinical symptoms become apparent but during which it is detectable by a screening test. Its clinical relevance is that it represents the duration of the temporal window of opportunity for early detection. The authors conduct a simple sensitivity analysis to determine the most important parameters in the model, which turn out to be the fraction of cases that are asymptomatic. Contrary to simple SLIR (or SEIR) models, this model allows to consider infection by asymptomatic individuals. Their predictions are over the short term, about 1 month. Other works, such as (23), also hone on identifying the unreported asymptomatic infectious cases (in mainland China). Their objective is to identify numbers for these individuals from specific time data of reported symptomatic infectious cases.

Also from the Centre for the Mathematical Modeling of Infectious Diseases COVID-19 working group, paper (31) tackles the problem of contact matrices. This work updates synthetic contact matrices that were published for Europe in 2017, with the most recent data and extends this analysis to 177 geographical locations. These matrices were constructed based on information that is more widely available than diary-based contact surveys and considers setting-specific survey data on household, school, classroom, and workplace composition combined with empirical data on contact patterns in Europe.

Authors in (32) discuss the “fundamental social causes” of disease, a factor that was up until now largely neglected when analyzing and predicting the effectiveness of prevention and mitigation measures. They argue that “inequitable social conditions lead to both more infections and worse outcomes” and expand the definition of “most at risk” to prioritize populations with social conditions and thus obtain more effective control of the epidemic.

The European Centre for Disease Prevention and Control (ECDC) (16) has built a Monte-Carlo based model of COVID that they use for forecasting. To model the behavior of the people and how well they are responding to the measures, they compare the predictions with Google data about mobile phone use. The most recent data on daily confirmed COVID-19 cases and daily deaths are inserted into the model to calibrate it. It currently works well for some countries but not for others.

There exist other COVID simulators based on the SEIR model (33), but they compute the number of infected, recovered, and dead individuals based on a mathematical model—solving the differential equations with a forward Euler scheme—on the basis of the number of contacts, probability of disease transmission, incubation period, recovery rate, and fatality rate. More complex versions of the SEIR model include, for instance, a quarantine class and a class of isolated (hospitalized) members (34). In (35), the authors use spatial diffusion of the virus, an alternative to contact networks. De la Sen et al. (36) propose an SEIADR model, where A are asymptomatic infectious and D are dead-infective. In other models, recovered can become susceptible again [e.g., (37)], and, in addition, there are stochastic models (38), although the calibration becomes extremely difficult with incomplete data.

In (39), the authors hypothesize—and discover evidence for—the order of symptom occurrence in COVID-19 vs. other respiratory diseases, to help patients and medical professionals more quickly distinguish between them. Although orthogonal, these findings may be used to improve testing and filter cases to more precisely record actual infections by Covid—in those cases where testing is not an option. This would give us more precise data to test on, which may help to calibrate our tool more precisely.

In general, simulation approaches based on agents are able to model the spread of infections more realistically and in detail, although they tend to suffer from scalability problems. We discuss a few of these approaches below. OpenABM-Covid19 (40) explores different ways in which contact tracing, in particular digital contact tracing via mobile phone apps can contribute to epidemic control, while emphasizing larger population simulations and computational efficiency. CPU time is spent mostly on rebuilding the daily interaction networks and updating the individual's interaction diaries. Individuals move daily between networks representing households and either workplaces, schools, or regular social environments for older people. The occupation networks are modeled as small-world networks. Individuals also interact through random networks representing public transport, transient social gatherings etc. Network parameters are chosen such that the average number of interactions match age-stratified data from reports. The current version of the model does not currently include events in hospitals, care-home settings, non-hospital deaths, gender, or co-morbidities. Different from us, the authors create daily contact networks based on actual mobile phone data. Occupation networks, on the other hand, are assumed to be of the small-world type and they are created as such rather than from interconnection patterns.

Covasim (41) includes demographic information about age structure and population size; realistic transmission networks in different social layers, including households, schools, workplaces, and communities; age-specific disease outcomes; and intra-host viral dynamics, including viral-load-based transmissibility. In terms of the contact network they use, Covasim is capable of generating three alternative types: random networks, SynthPops networks, and hybrid networks; in addition users have the option of defining their own networks. The SynthPops algorithm first chooses a reference individual for the specific layer, e.g., a school, to infer the school type, and then uses age the mixing contact matrix in the school setting to infer the likely ages of the other students in the school. Students are drawn from an ordered list of households. To deal with scalability issues, once a certain threshold in population number is reached, the non-susceptible agents in the model are downsampled and a corresponding scaling factor is introduced. Calibration to existing time-series data is performed externally to Covasim. One difference from our work is that the contacts are not based on existing patterns; scalability issues are partly sidestepped by dynamic scaling. On the other hand, their approach models critical patients, hospital capacity and ICU beds.

In (42), the authors integrate anonymized, geolocalized mobility data with census and demographic data to build a detailed agent-based model of Covid-19 transmission in the Boston metropolitan area. Their approach defines a weighted network with layers for the network of social interactions at (1) workplace and community level, (2) households, and (3) schools. Connections between two agents in the workplace and community layer are estimated from the data by the probability of both being present in a specific place weighted by the time they have spent in the same place. They do not include specific co-morbidities or pre-existing conditions of the specific population. Different from us, the authors construct the interaction network based on co-location at the same time, starting from mobility data. Interactions are considered well-mixed in school environments, while in our simulator every individual has its own characteristics and interaction patterns.

The work in (43) calibrates the simulator based on daily ICU admissions, ICU-bed occupancy, daily mortality and cumulative mortality. They approximate the value for the R0 from the observed average number of new individuals infected by each single infected individual from the beginning of the epidemic until about 30 days after. The numbers of infected and infecting people were estimated using the model. We, on the other hand, use the R0s from the literature for each of the infection phases. One of their conclusions is that, in the absence of a vaccine, emphasis should be placed on policies that protect the most-vulnerable population while herd immunity is hoped to be achieved in the less vulnerable people. The social contact network among the individuals in the population is based on the geo-localized activity sequence over the day, taking into account co-location probability and duration, another difference with our work.

Koo et al. (44) uses FluTE, an agent-based influenza epidemic simulation model, which accounts for demography, host movement, and social contact rates in workplaces, schools, and homes. Individuals are allocated to workplaces or educational facilities on the basis of local transportation data and home addresses according to 2010 census data in Singapore. They conclude that spread control is feasible provided that R0 is low (≤ 1.5), with a combination of quarantine, school closure, and workplace distancing, assuming a low percentage of asymptomatic of 7.5% The preventive effect of these interventions reduces considerably when higher asymptomatic proportions are assumed (all under 50%), when quarantining and treatment of infected individuals becomes more important and also unfeasible when the number of infected individuals exceeds the capacity of health-care facilities. This work relies on detailed data in Singapore, which is feasible given the size and infrastructure of the country. The study focuses on the types of measures that enable controlling the infection spread, although the values they use for some of the parameters seem unrealistically small.

Lau et al. use in (45) a dataset that contains demographic information of 9,559 symptomatic cases in Georgia, US, including age, sex, and race, and symptom onset times. It also contains geo-location information of the residences of all these recorded cases. Aggregate mobility data are used to characterize the average change of movement distance before and after the implementation of social distancing measures. Their framework infers the transmission paths among all cases and therefore generates the offspring distribution of each case; it also allows the computation of population-level epidemiological parameters such as R0 and quantify the degree of super-spreading over space and time. This is a very different type of work from ours, as it relies on concrete and detailed data from about 10K cases, based on which the authors can tackle the issue of superspreaders.

The work presented in (46) uses an ABM model specifically calibrated to reproduce the reproductive number, the length of incubation and generation periods, age-dependent fractions of the symptomatic cases and the probability of transmission from asymptomatic/pre-symptomatic agents in Australia. Contact and transmission rates were set to differ across distinct social contexts such as households, household clusters, local neighborhoods, schools, classrooms and workplaces. To infer a directed transmission link from the simulation results, the model connects any two infected individuals with the same household identifier, same neighborhood (household cluster) label or same wider community (SLA) index. The authors compare the findings using the ABM with genomic surveillance based on near real-time genome sequencing of Covid-19 in a sub-population of infected patients during the first 10 weeks of containment in Australia. The approach has limitations, e.g., the genomic study did not describe transmissions from asymptomatic carriage, while the ABM did not explicitly simulate transmissions in hospitals, residential age care facilities, or introduced by maritime traffic, for example, cruise ships. The results compare favorably, but including genomic sequencing allows to find potential sources of infections that cannot be identified using conventional epidemiological methods. Differently from other work we have seen, this work relies on genomic surveillance data to confirm the results obtained by their ABM simulator.

In (47), Silva et al. emulated a closed society living on a shared environment, consisting of agents that represent people, houses, businesses, the government and the healthcare system, each one with specific attributes and behaviors. The ABM proposed by the authors also models the economy in this society of agents, which helps them estimate the economic impact under different types of interventions. Their model considers that a contact happens when the distance between any two agents is less than or equal to a defined threshold; this contact can be epidemiological or economical. This is a rare example of an approach that also models the behavior of other entities that are not individuals and thus can help understand the impact of the pandemic both on citizens and the economy.

## 6. Conclusion

This work was developed in the context of the project *Medium and Long-term Simulation of Covid-19* funded by the Spanish Health Ministry. The results we obtained provided support to the health authorities for the forecasting of the first wave and in the evaluation of possible future scenarios. The work targets the metropolitan area of Madrid, which we model in detail to take into account social aspects such as age distribution and occupation, size of family units, percentage of workers, school children, unemployed, and stay home parents. The epidemiological model is updated with the COVID-specific values, including the R0s of each state and the time spent in each infection phase, as well as the probabilities for hospitalization and death depending on age (which we use in conjunction with existent mortality data for calibration of the simulator).

The main conclusions of this work are that EpiGraph is able to reproduce both the existing infected and death curves for Madrid metropolitan area in the Spring 2020. Regarding the elderly confinement, this social distancing policy would help to lower the number of deaths (not the number of infections), which, although reduced, would have remained important. We also evaluated that school opening after the lock down, which has a minor impact on the infection spread. In terms of mask use, the percentage of people that comply becomes a crucial factor for mitigating the infection spread. In the longer term, our tool could help to plan for a more resilient and efficient approach to epidemics, and study how to flexibly respond and adapt to this type of unexpected situations.

## Data Availability Statement

The dataset supporting the conclusions of this article is available in the repository (10.3389/fpubh.2021.636023).

## Author Contributions

DS, M-CM, and JC designed and implemented EpiGraph simulator. All authors conceived and designed the experiments. DS, MG-M, and CD processed the input data and ran the experiments. DS, JC, and M-CM wrote the paper. CD-S and DG-B provided insights on the validity of our assumptions, recommended additional related work, and contrasted the results with their own findings. All authors review the different manuscript drafts and approved the final version for submission.

## Conflict of Interest

The authors declare that the research was conducted in the absence of any commercial or financial relationships that could be construed as a potential conflict of interest.

## Footnotes

^1^National Statistics Institute (INE). Available online at: http://www.ine.es/ (2021).

^2^We define a social contact (also called contact or interaction) between two individuals a co-location in time (and space) at a distance that is small enough to make transmission possible.

^3^This time interval is an average value obtained from public and private school timetables, and takes into account that some students leave school before lunch while others stay on during the afternoon.

^4^For groups with different sizes, the maximum number of available inter-group connections is the size of the smaller group. For instance, two groups with 50 and 100 individuals that are strongly connected with 90% contacts, will have 45 inter-group connections between them.

^5^Google Maps API. Available online at: https://developers.google.com/maps (2021).

^6^Note that here we are not considering the *E*^*P*^ stage because we assume that individuals in this stage have not developed symptoms.

^7^Red Nacional de Vigilancia de la Salud Publica. Available online at: https://cnecovid.isciii.es/covid19/ (2021).

^8^In the figures, we have represented the values using the same time step (for the x-axis values) used by the simulator (10 min). This makes the graphics more detailed and smooth.
