# Corrigendum: Simulation of COVID-19 propagation scenarios in the Madrid metropolitan area

**DOI:** 10.3389/fpubh.2023.1180932

**Published:** 2023-03-16

**Authors:** David E. Singh, Maria-Cristina Marinescu, Miguel Guzmán-Merino, Christian Durán, Concepción Delgado-Sanz, Diana Gomez-Barroso, Jesus Carretero

**Affiliations:** ^1^Department Computer Science, Universidad Carlos III de Madrid, Leganés, Spain; ^2^Barcelona Supercomputing Center, Barcelona, Spain; ^3^CIBER en Epidemiología y Salud Pública (CIBERESP), Madrid, Spain; ^4^National Centre for Epidemiology, Carlos III Institute of Health, Madrid, Spain

**Keywords:** COVID-19, simulation, social distancing, mitigation policies, face mask

In the published article, there was an error in the **Funding** statement. The project grant reference 2020/00183/001 is wrong. The correct **Funding** statement appears below.

