# Supplementary material for: Simulation of COVID-19 Propagation Scenarios in the Madrid Metropolitan Area
Source: Front Public Health. 2021 Mar 16;9:636023. doi: 10.3389/fpubh.2021.636023 (PMC8007867; doi:10.3389/fpubh.2021.636023)
Supplement: Supplementary file 1 [file Data_Sheet_1.PDF]

Supplementary Material

1 SUPPLEMENTARY TABLES AND FIGURES

1.1 Tables

School groups					
MinAge	0	MaxAge	19	Percentage	0.1757 %
MinSize	40	MaxSize	200	Percentage males	0.5108 %
Work groups					
MinAge	20	MaxAge	64	Percentage	0.5179 %
MinSize	20	MaxSize	1000	Percentage males	0.4770 %
Stay-at-home, informal meetups groups					
MinAge	20	MaxAge	64	Percentage	0.1194 %
MinSize	1	MaxSize	10	Percentage males	0.4770 %
Elder, informal meetups groups					
MinAge	65	MaxAge	100	Percentage	0.1870 %
MinSize	25	MaxSize	50	Percentage males	0.3905 %

Table S1. Social group distribution for the cities of Madrid metropolitan area. The percentage of companies that open on Saturday are 35 %.

Number of members in a family					
1 member	25.50 %	2 members	30.40 %	3 members	20.90 %
4 members	17.70 %	5 members	5.50 %		

Table S2. Family size distribution for the cities of the Madrid metropolitan area.

1.2 Figures

Worker, student, stay-at-home and elder activity cycles for weekends and Holidays.

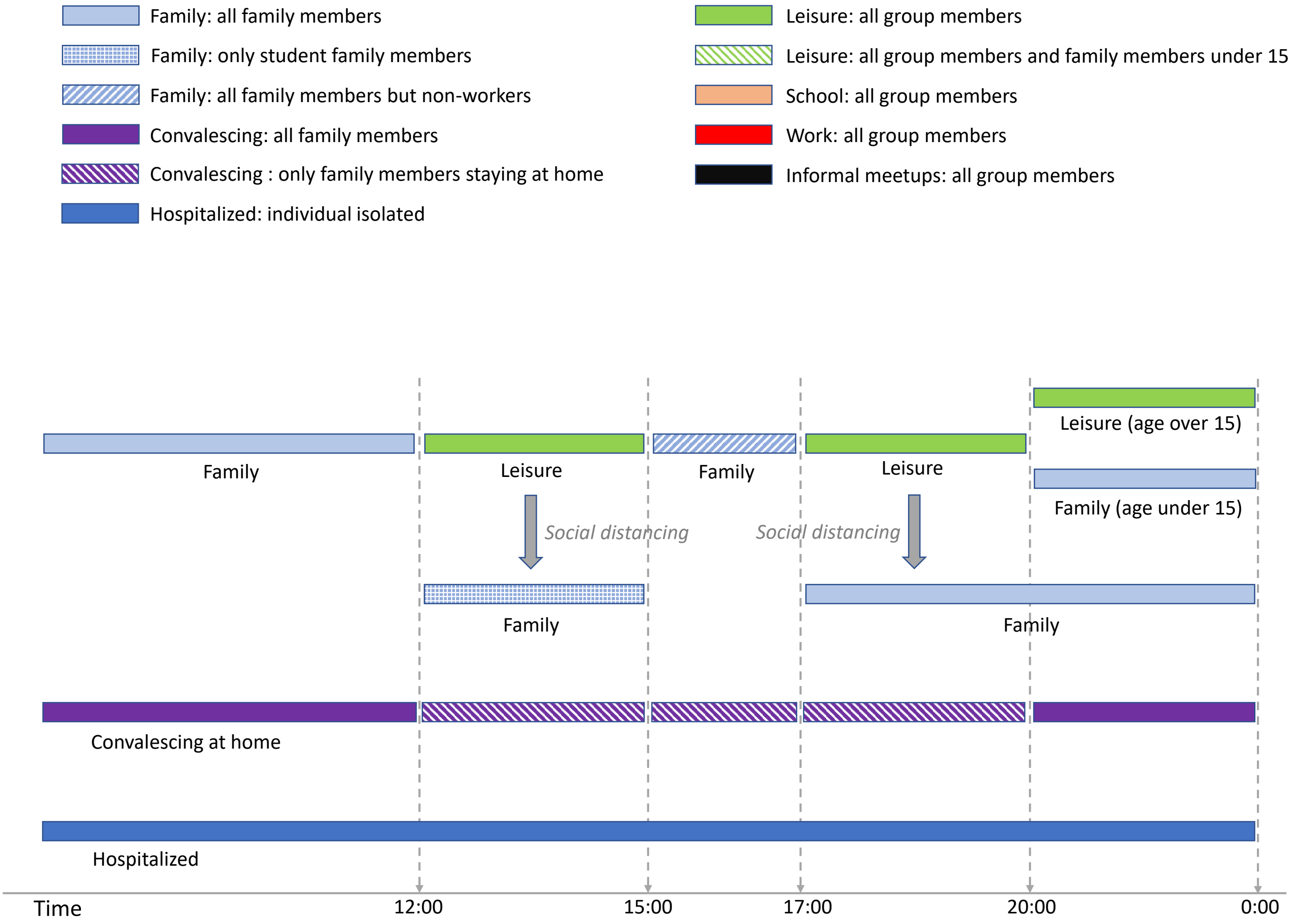

Figure S1. Student activity cycle for Saturday. The interaction patterns are specific for Spain. Night time is considered to extend from midnight to 9:00 and is not included in the figure (but is considered in the simulation). The text in italic shows the effect of social restrictions. School time is an average of the public and private timetables.

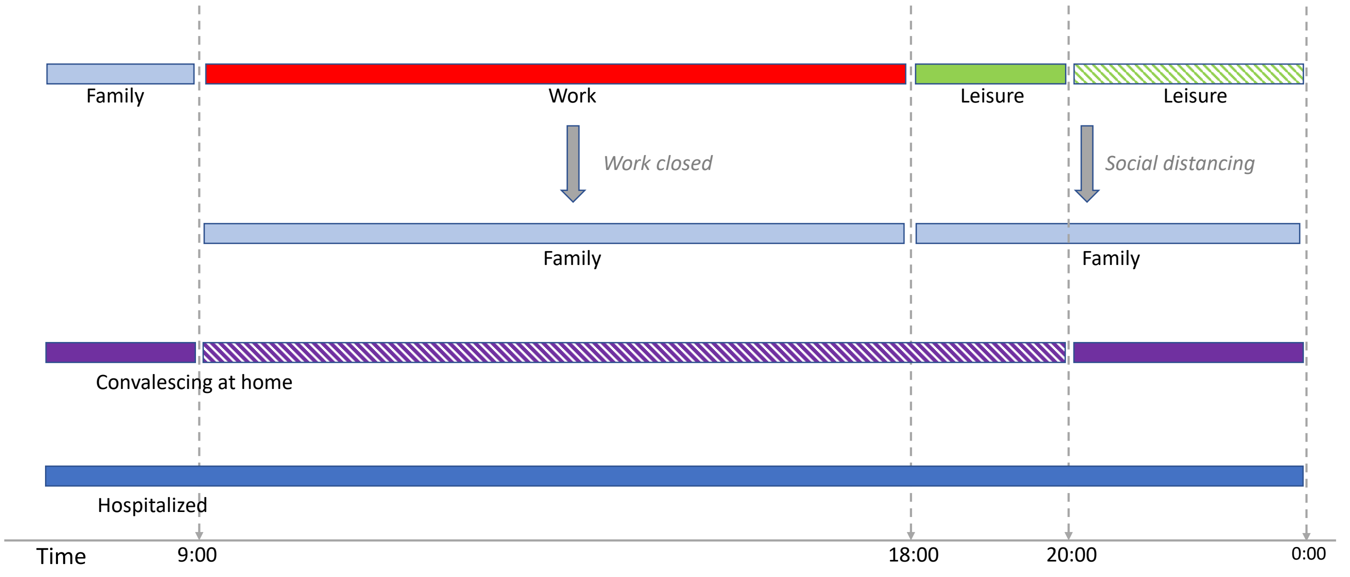

Figure S2. Worker activity cycle for workers that work on Saturday. This collective represents the 35% of the total workers. The interaction patterns are based on Spanish culture. The interaction patterns are specific for Spain. Night time is considered to extend from midnight to 9:00 and is not included in the figure (but is considered in the simulation). The text in italic shows the effect of social restrictions.

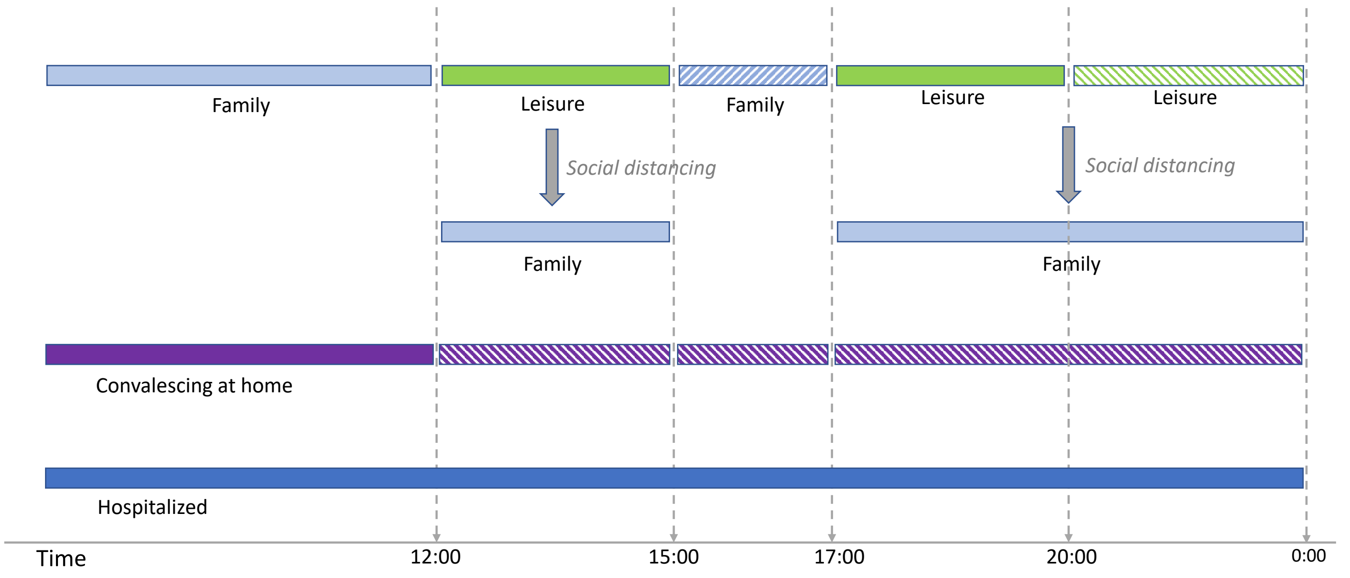

Figure S3. Worker activity cycle for workers that do not work on Saturday. This collective represents the 65% of the total workers. The interaction patterns are based on Spanish culture. The interaction patterns are specific for Spain. Night time is considered to extend from midnight to 9:00 and is not included in the figure (but is considered in the simulation). The text in italic shows the effect of social restrictions.

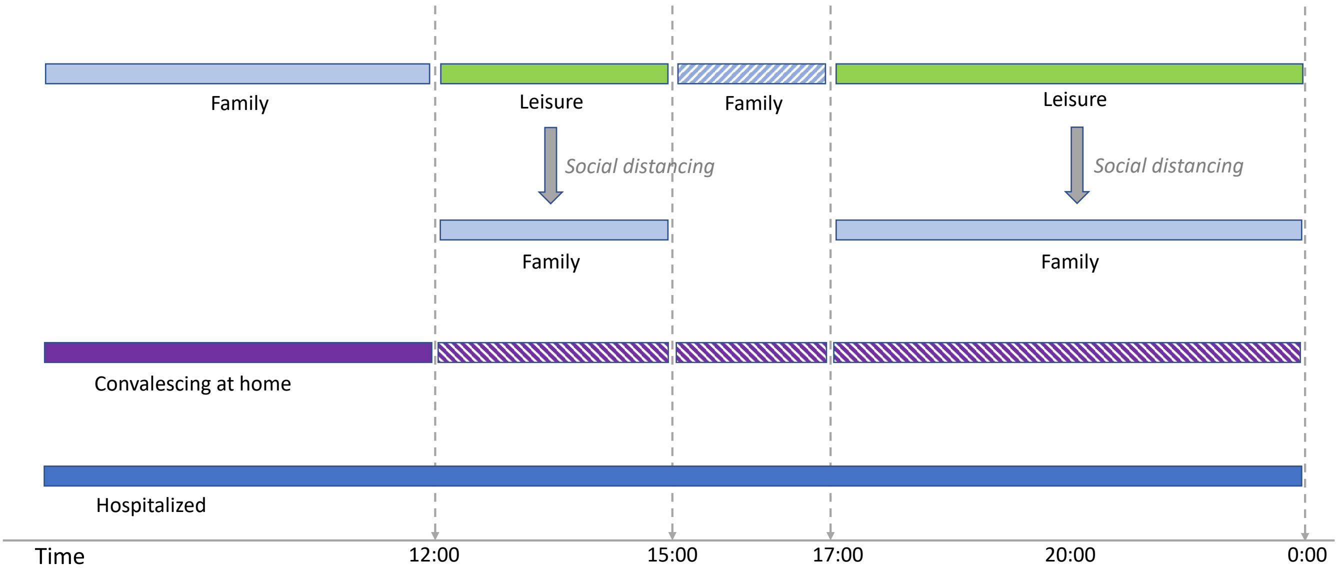

Figure S4. Stay-at-home activity cycle for Saturday. The interaction patterns are based on Spanish culture. The interaction patterns are specific for Spain. Night time is considered to extend from midnight to 9:00 and is not included in the figure (but is considered in the simulation). The text in italic shows the effect of social restrictions.

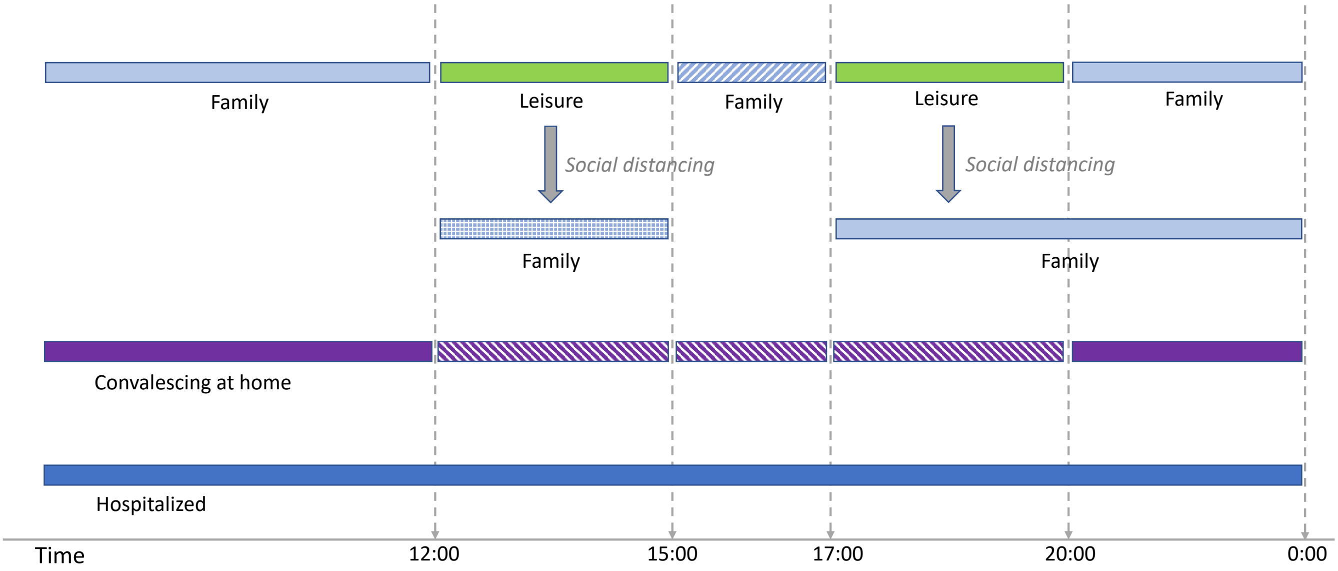

Figure S5. Elder cycle for Saturday, Sunday and holidays. The interaction patterns are based on Spanish culture. The interaction patterns are specific for Spain. Night time is considered to extend from midnight to 9:00 and is not included in the figure (but is considered in the simulation). The text in italic shows the effect of social restrictions.

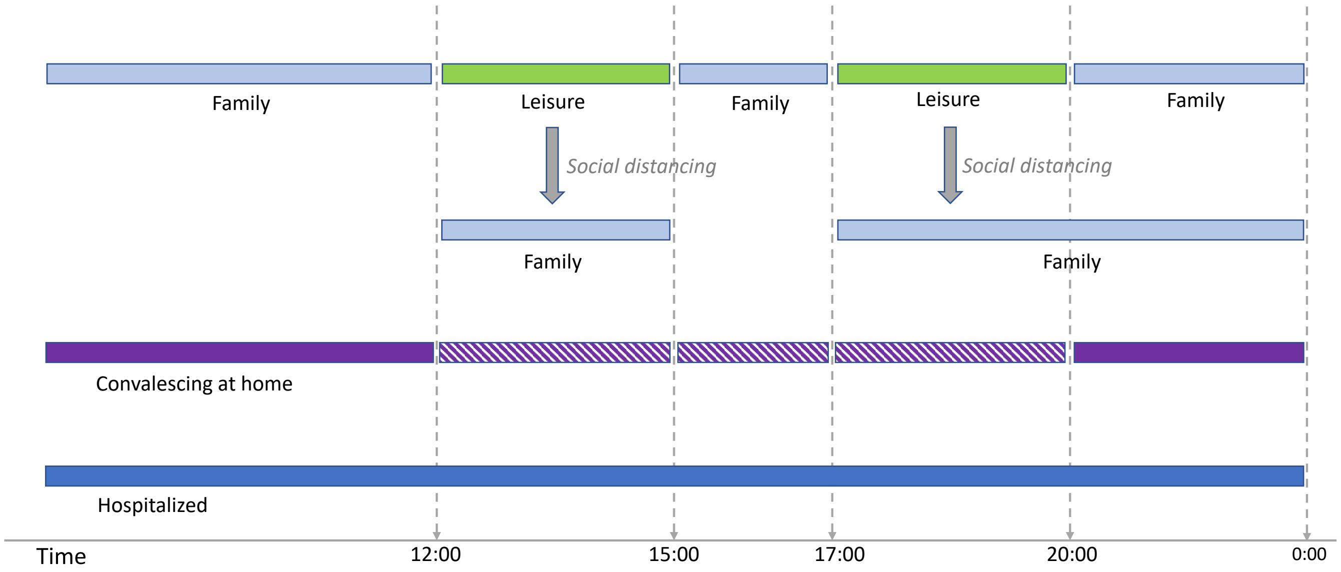

Figure S6. Student activity cycle for Sunday and holidays. The interaction patterns are based on Spanish culture. The interaction patterns are specific for Spain. Night time is considered to extend from midnight to 9:00 and is not included in the figure (but is considered in the simulation). The text in italic shows the effect of social restrictions.

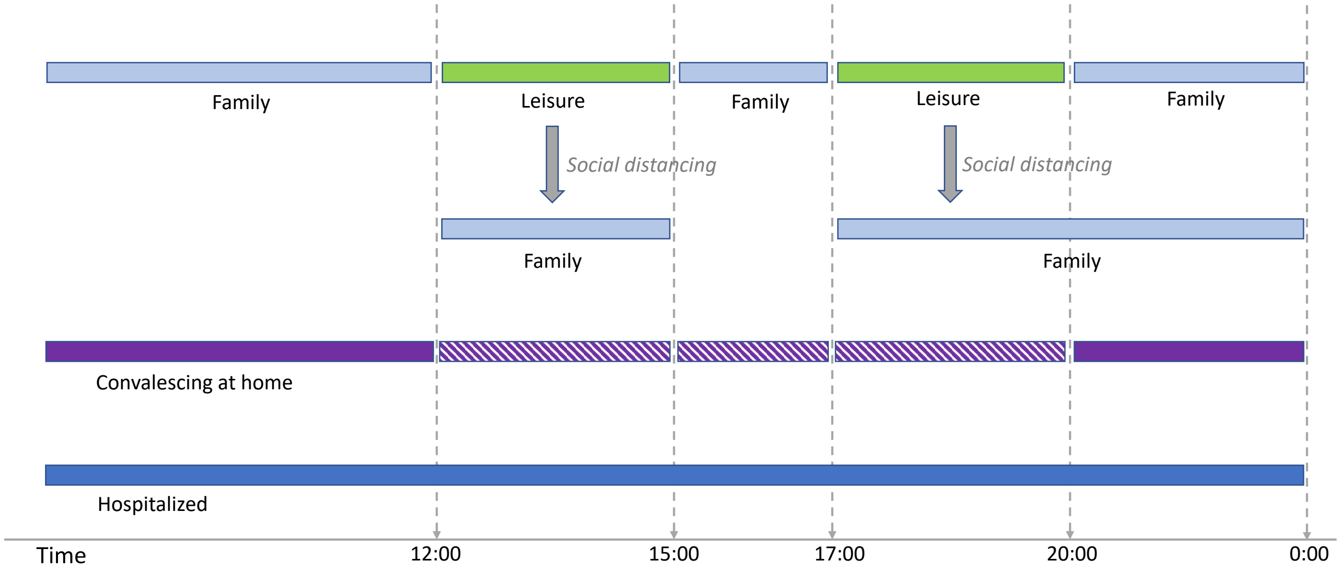

Figure S7. Worker activity cycle for Sunday and holidays. The interaction patterns are based on Spanish culture. The interaction patterns are specific for Spain. Night time is considered to extend from midnight to 9:00 and is not included in the figure (but is considered in the simulation). The text in italic shows the effect of social restrictions.

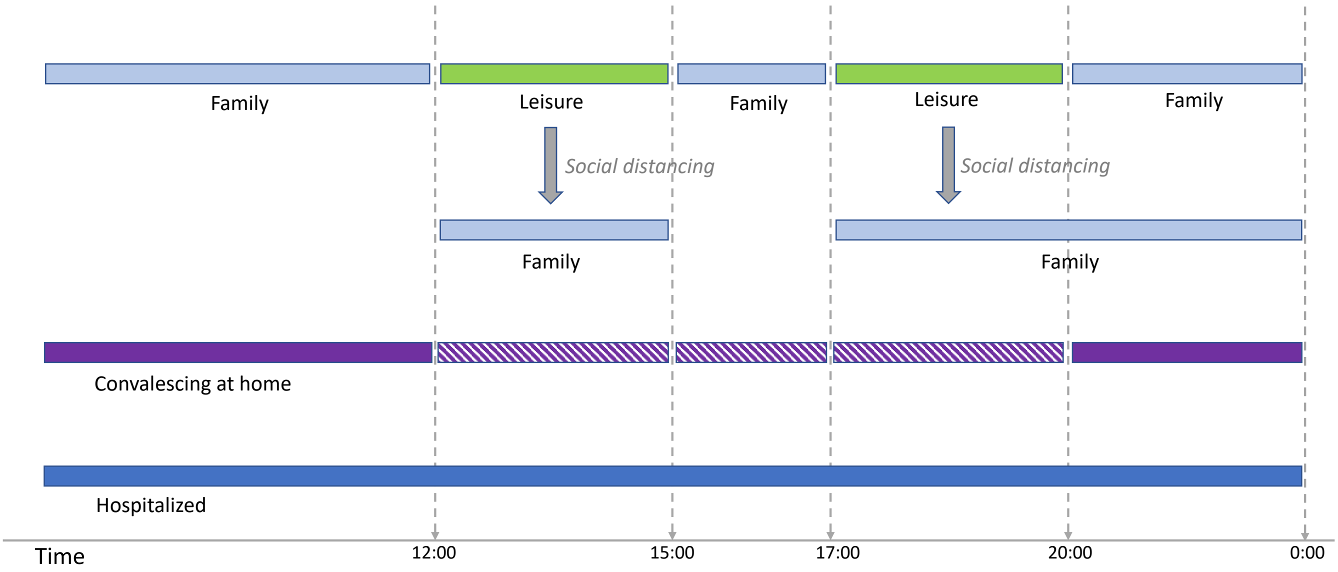

Figure S8. Stay-at-home activity cycle for Sunday and holidays. The interaction patterns are based on Spanish culture. The interaction patterns are specific for Spain. Night time is considered to extend from midnight to 9:00 and is not included in the figure (but is considered in the simulation). The text in italic shows the effect of social restrictions.
